# Mapping the prevalence of the neglected sexual side effects after prostate cancer treatment and the questionnaires used in their screening: a scoping review protocol

**DOI:** 10.1186/s13643-020-01473-9

**Published:** 2020-09-17

**Authors:** Pierre Röscher, Jacqueline M. van Wyk

**Affiliations:** Nelson R. Mandela School of Medicine, University of KwaZulu-Natal, 719 Umbilo Rd, Umbilo, Berea, 4001 South Africa

**Keywords:** Prostate cancer, Prevalence, Questionnaire use, Screening tool, Orgasm-associated incontinence, Urinary incontinence during sexual stimulation, Altered perception of orgasm, Orgasm associated pain, Penile shortening, Penile deformity

## Abstract

**Background:**

Interventions to treat early prostate cancer (PCa) can leave men with debilitating sexual side effects. The cluster of side effects referred to as the neglected sexual side effects (NSSE) may remain permanent, undiagnosed and untreated because men are hesitant to disclose them. Questionnaires offer a discreet way into the discussion, subsequent diagnosis and possible treatment of the NSSE. This study will be conducted to map the evidence about the prevalence of the neglected sexual side effects (NSSE) after PCa treatment, and use of questionnaires in its diagnosis and screening.

**Methods:**

This systematic scoping review will involve searching the following electronic databases: PubMed, Science Direct and Google Scholar. Following title searching, two-independent reviewers will conduct screening of abstracts and full articles. Eligibility criteria will guide the screenings. Data will be extracted from the included studies, and the emerging themes will be analysed. The review team will analyse the implications of the findings concerning the research question and aim of the study. The mixed method appraisal tool (MMAT) will be employed for quality appraisal of included studies.

**Discussion:**

We anticipate finding a number of studies that describe the prevalence of NSSE after early PCa treatment and that report on using questionnaires to screen for the presence of symptoms including orgasm-associated incontinence, urinary incontinence during sexual stimulation, altered perceptions of orgasm, orgasm associated pain, penile shortening and penile deformity. The study findings will be disseminated through publication in a peer-reviewed journal, peer presentations and presentations at relevant conferences.

## Background

Prostate cancer (PCa) is a significant cause of disease and mortality amongst men, and it is the second most common cancer affecting men on a global scale [1]. Early PCa or localised PCa is cancer contained within the prostate described as being stage I or II on the tumour-node-metastasis system [2]. Early PCa treatment consisting of surgery or radiotherapy, either through external beam radiotherapy or brachytherapy, results in side effects including sexual dysfunction. Other common side effects could include both pain and incontinence [1]. Sexual dysfunction from PCa treatment is common regardless of whether the treatment modality included surgical or non-surgical interventions. Studies suggest that sexual dysfunction increase during each year of follow-up after the initial intervention, and it affects an average of 50% of patients within 5 years of receiving treatment [3].

Most men generally recover from pain and incontinence after PCa surgery, but sexual dysfunction often remains untreated, leaving them with long-lasting and devastating sexual dysfunction [1]. Specific conditions related to sexual dysfunction are common after PCa treatment. The conditions include orgasm-associated incontinence, urinary incontinence during sexual stimulation, altered perception of orgasm, orgasm associated pain, penile shortening and penile deformity [1, 4, 5]. These conditions are collectively referred to as the ‘Neglected Sexual Side Effects’ (NSSE), and the symptoms are reportedly prevalent in 20–93% of post-prostatectomy patients [1].

Only a fifth of the men who are diagnosed with PCa will ever discuss issues relating to sexual dysfunction with their health care practitioners (HCP) [6]. A questionnaire may provide a non-threatening strategy to initiate such a discussion and allow the patient to indicate their presenting symptoms. Two validated questionnaires, the expanded prostate cancer index composite (EPIC) [7] and the international index of erectile function (IIEF) [8], were recommended for use in this context in 2015 [9].

### Reason for this review

Whilst the EPIC and IIEF both help to stimulate the conversation around general urinary and sexual function, they do not address the NSSE after PCa treatment. There is a need to map the evidence about the use of a questionnaire to help health care providers screen for any of the NSSEs after PCa treatment. It is therefore essential to conduct a systematic scoping review to improve our understanding of the prevalence of NSSE and to highlight knowledge gaps on the role of questionnaires in diagnosis and screening of the NSSEs.

## Methodology

A systematic scoping review will be conducted to map the evidence on (i) the prevalence of NSSEs after early treatment PCa and (ii) summarise the literature on the use of questionnaires in the screening of NSSE after early treatment for PCa.

The scoping review will follow the five steps described by Arksey and O’Malley [10] that include the following:

1. Identifying the research question
2. Identifying relevant studies
3. Study selection
4. Charting the data
5. Collating, summarising and reporting on the data

Quality assessment of each of the included primary studies will be done as guided by Levac et al. [11].

### Identifying the research question

This review aims to identify current academic literature on the NSSE after men have undergone early treatment for PCa. This early treatment includes radical prostatectomy surgery and radiation therapy.

The research questions are as follows:

What is the prevalence of NSSE after early treatment for PCa?

Which questionnaires are being used to assess NSSE after early treatment for PCa?

### Identifying relevant studies

A search will be conducted for published and unpublished (grey) literature to identify eligible studies in the following electronic databases: PubMed, Science Direct and Google Scholar databases. We will also include relevant studies found in citations and reference lists of included articles. The search will include publications available in English and published between January 2009 and December 2019.

### Eligibility criteria

The population concept context (PCC) framework will inform the eligibility of the research question, as illustrated in Table 1.

**Table 1:** The PCC framework

	Criteria	Determinants
P	Population	Men who received surgical and non-surgical treatment following early PCa diagnosis
		• Surgical treatment (radical prostatectomy surgery)
		• Non-surgical treatment (radiation therapy)
C	Concept	Neglected sexual side effects (NSSE)
		• Anejaculation
		• Orgasmic pain
		• Orgasmic dysfunction
		• Climacturia
		• Urinary incontinence from sexual stimulation
		• Peyronies disease
		• Penile length shortening
C	Context	Prevalence of NSSE
		Questionnaires used to screen for the prevalence NSSE

Boolean terms (AND, OR) and Medical Subject Headings (MeSH) will be used, as indicated in Table 2. The search results will be captured on an Excel spreadsheet where the duplicates will be removed. The selected studies will be screened against the eligibility criteria. The study search strategy was piloted to determine the appropriateness and feasibility of conducting this study, and the results are presented in Table 2.

**Table 2:** Pilot database search results

Keyword search	Date of search	Search engine used	No. of publications retrieved
(Orgas* OR Penil* OR Climacturia (MeSH Terms) OR Dysorgasmia (MeSH Terms) OR anejaculation (MeSH Terms) OR Peyronie OR neglected AND [prostate cancer (MeSH Terms) OR Prostatectomy (MeSH Terms)]	1 September 2019	Pubmed	152

### Selection of eligible studies

A set of eligibility criteria was developed to ensure that the included studies are relevant to address the research question. The results of the databases will be combined into one Excel spreadsheet after applying the search parameters. The eligibility criteria were developed to ensure that selected studies contain relevant information to answer the review questions.

The study’s inclusion and exclusion criteria are summarised in Table 3.

**Table 3:** Inclusion and exclusion criteria of the study

The inclusion criteria	The exclusion criteria
Only primary studies that present evidence on the following:	• Review articles
• The prevalence of NSSE after early stage PCa treatment	• Non-peer reviewed articles (e.g. books, magazines, policy briefs)
• The use of questionnaires to screen for the prevalence of NSSE after early stage PCa treatment	• Commentaries, editorials, programme evaluations and letters
	• Publications on sexual dysfunction not relating to the prevalence and the use of questionnaires to screen for NSSE after early PCa treatment
• Original studies available in English and published between 1 January 2009‑31 December 2019	
	• Studies outside the period of interest and studies not available in English

The primary investigator will conduct a comprehensive search and screening of the study titles from the databases, as mentioned above. All the relevant studies with appropriate titles will be extracted and entered into an Excel spreadsheet for processing. All articles that cannot be extracted will be requested from the University of KwaZulu Natal library services, or the authors will be contacted via email. All duplicates will be removed before the titles are screened. Two reviewers will review the abstracts of the eligible studies. The principal researcher and a medically trained research assistant will each conduct an independent full-text screening. The inclusion and exclusion criteria will be applied to identify the qualifying articles. The inter-rater agreement (Cohen’s kappa coefficient (*k*) statistic) between reviewers will be calculated after full-text screening [12].

Any discrepancies in reviewers’ results during the abstract and full-text screening stage will be resolved through discussion until agreement is reached. If needed, a third reviewer will be used to settle discrepancies. The screening result will be reported using the Preferred Reporting Items for Systematic Reviews and Meta-Analyses (PRISMA) chart [13].

### Charting the data

The information will be extracted and organised using a data charting form. Data will be processed so that the relevant information can be summarised to answer the research questions. The data charting tool, as illustrated in Table 4, will be used by a second reviewer to validate all the information.

**Table 4:** Data charting form

Author, date and reference Aims and research questions Geographical setting Study population Study design Number of participants Period post-PCa investigated Prevalence of NSSE Reported use of questionnaire to screen for NSSE after PCa Quality of the study	

### Quality appraisal

An electronic version of the mixed method appraisal tool (MMAT) [14] will be adapted to assess the quality of the included studies. The study designs included in this scoping review will include qualitative, quantitative descriptive and mixed methods studies. The specific criteria to determine the appropriateness of each included study are outlined in Appendix.

Two reviewers will assign a score to assess each article that will assess the appropriateness of the study aims and its relevance for inclusion on the review. The overall quality for each included study will be calculated according to the following MMAT guidelines (score = number of criteria met/total score in each domain). One point will be given for each question, and a total score out of 5 will be calculated. The calculation will be presented as a percentage which correlates to the degree to which the identified was assessed to provide relevant information to answer the research question (Appendix).

The results will use the following descriptors.

- Very poor quality (20%) where minimal criteria are met
- Poor quality (40%) where less than half the criteria are not met
- Fair quality (60%) where just more than half the criteria are met
- Good quality (80%) where most of the criteria are met
- Excellent quality (100%) all criteria are met

The overall quality of a combination of components cannot be more than its weakest component when it comes to mixed-methods studies, making the overall score equal to the lowest-scoring component [14].

### Collating, summarising and reporting on the data

The collected data will firstly be reported by using descriptive statistics about (i) the geographical setting of studies, (ii) study populations, (iii) study designs, (iv) number of participants, (v) period post-PCa investigated, (vi) prevalence of NSSE, (vii) reported use of a questionnaire and (viii) quality of the studies.

Secondly, the findings of this scoping review will be analysed using a content analysis approach of the themes emerging from the extracted data. The themes will be collated to answer each research question.

The review team will discuss findings, resolve issues, and finalise findings. The review team will analyse the implications of the findings in relation to the study aims and further research in the field.

## Discussion

PCa constitutes a global public health burden [15], and surgical and non-surgical interventions are routinely administered [16]. Men who receive treatment for early stage PCa are often unaware of the debilitating, long-lasting side effects following the treatment [4]. Sexual function has been identified as the quality of life domain most strongly associated with outcome satisfaction after prostate cancer treatment [17]. With most research in the field of PCa focused around incontinence and erectile dysfunction, the NSSE remains understudied and neglected [1, 18]. This review will report on the prevalence of the NSSE after early PCa treatment.

Only two studies have been published on the NSSE related to PCa treatment [5, 19]. There is also no current valid and reliable questionnaire being used in the field of the NSSE after early PCa treatment. Such a questionnaire would assist health care practitioners to screen for possible NSSEs in patients who had undergone treatment for early PCa.

A review of the literature related to the prevalence of the NSSE after PCa treatment and the questionnaires used to screen for them may help to inform future clinical practice around the NSSE in PCa survivors.

## Data Availability

Not applicable

## Appendix

Selection of MMAT questions: Specific criteria to determine the appropriateness for inclusion of each study

Qualitative, quantitative descriptive and mixed methods studies

The methodological quality criteria applied to evaluate qualitative studies included the folowing:

1. Is the qualitative approach appropriate to answer the research question?
2. Are the qualitative data collection methods adequate to address the research question?
3. Are the findings adequately derived from the data?
4. Do data sufficiently substantiate the interpretation of results?
5. Is there coherence between qualitative data sources, collection, analysis and interpretation?

The criteria to evaluate quantitative descriptive studies include the following:

1. Is the sampling strategy relevant to address the research question?
2. Is the sample representative of the target population?
3. Are the measurements appropriate?
4. Is the risk of non-response bias low?
5. Is the statistical analysis appropriate to answer the research question?

The criteria to evaluate mixed methods studies include the following:

1. Is there an adequate rationale for using a mixed-method design to address the research question?
2. Are the different components of the study effectively integrated to answer the research question?
3. Are the outputs of the integration of qualitative and quantitative components adequately interpreted?
4. Are divergences and inconsistencies between quantitative and qualitative results adequately addressed?
5. Do the different components of the study adhere to the quality criteria of each tradition of the methods involved?

Each study will be evaluated according to its study design based on the above criteria. One point was given for each question, and a total score out of 5 was calculated. This was represented as a percentage, which correlated to the quality of the included studies (Appendix). The principal investigator will perform each quality assessment.

## Abbreviations

PCa: Prostate cancer
HCP: Health care practitioner
NSSE: Neglected sexual side effects
EPIC: Expanded prostate cancer index
IIEF: International index of erectile function
MMAT: Mixed method appraisal tool
